# Comprehensive three-dimensional analysis of right-sided aortic arch with multiple vascular anomalies

**DOI:** 10.1186/1471-2261-14-104

**Published:** 2014-08-20

**Authors:** Chan-Hee Lee, Jang-Won Son, Jong-Seon Park

**Affiliations:** 1 Department of Cardiology, Yeungnam University Hospital, 170, Hyeonchungro, Nam-gu, Daegu 705-717, Korea

**Keywords:** Right-sided aortic arch, Kommerell’s diverticulum, 3-dimensional reconstruction

## Abstract

**Background:**

Right-sided aortic arch is a rare congenital defect usually diagnosed incidentally in adults; it is often asymptomatic unless aneurismal disease develops. In half the cases, an aberrant left subclavian artery arises from a Kommerell’s diverticulum; in these cases, congenital heart anomaly is very rarely present.

**Case presentation:**

We report a case of incidentally-detected right-sided aortic arch with multiple vascular anomalies including left subclavian artery originating from a Kommerell’s diverticulum, supra-sinus origin of coronary arteries and coronary arteriovenous fistula.

**Conclusion:**

Through comprehensive 3-dimensional reconstruction of the aortic arch and surrounding structures we defined anatomical relationships, which is useful for follow-up and treatment.

## Background

In 1936, Burckhard Friedrich Kommerell first described an aberrant right subclavian artery originating from the descending thoracic aorta of a left-sided arch and associated with persistence of a remnant of the right dorsal aorta. This now called Kommerell’s diverticulum consists of both an aneurysm of the thoracic aorta and an aneurismal orifice of the aberrant subclavian artery [1]. Right-sided aortic arch with aberrant left subclavian artery originating from a Kommerell’s diverticulum is an uncommon arch abnormality seen in about 0.05% of the general population [2]; it is usually asymptomatic and incidentally detected. Symptoms in adults often result from early atherosclerotic changes in the anomalous vessels, and/or dissection or aneurismal dilatation with compression of surrounding structures [3]. Computed tomography (CT) now allows three-dimensional (3D) reconstruction of multiple organs, as exemplified in this case report of a patient with right-sided aortic arch and multiple vascular anomalies. This information is relevant to symptom follow-up and treatment.

## Case presentation

**Figure 1:**
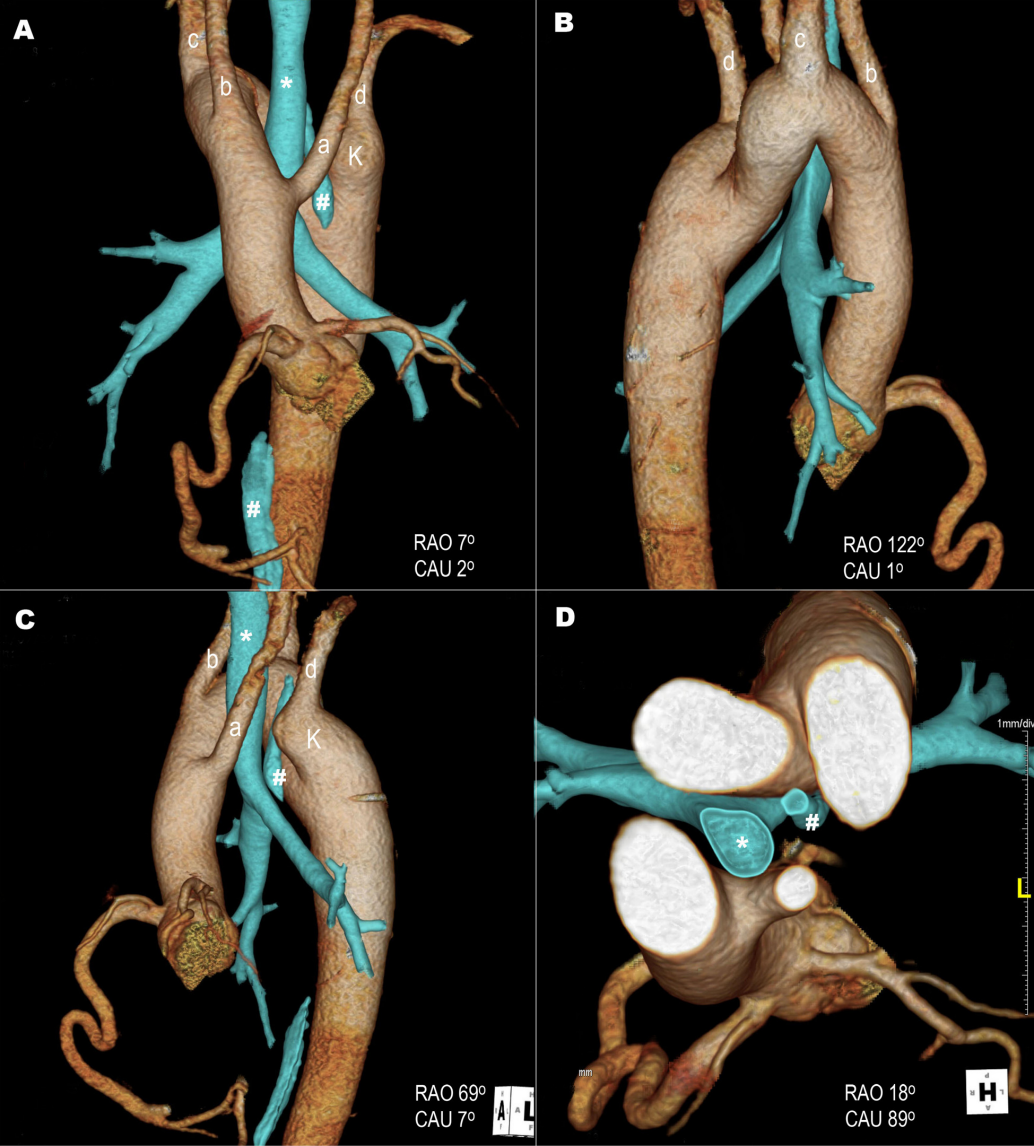
**Three-dimensional CT angiography images.** Relationships of right-sided aortic arch and its branches with trachea (*) and esophagus (#) in different directions **(A-D)**. Left common carotid artery (a), right common carotid artery (b) and right subclavian artery (c), originated separately in said order. Left subclavian artery (d) separately arises from a Kommerell’s diverticulum (K). RAO indicates right anterior oblique; CAU, caudal.

**Figure 2:**
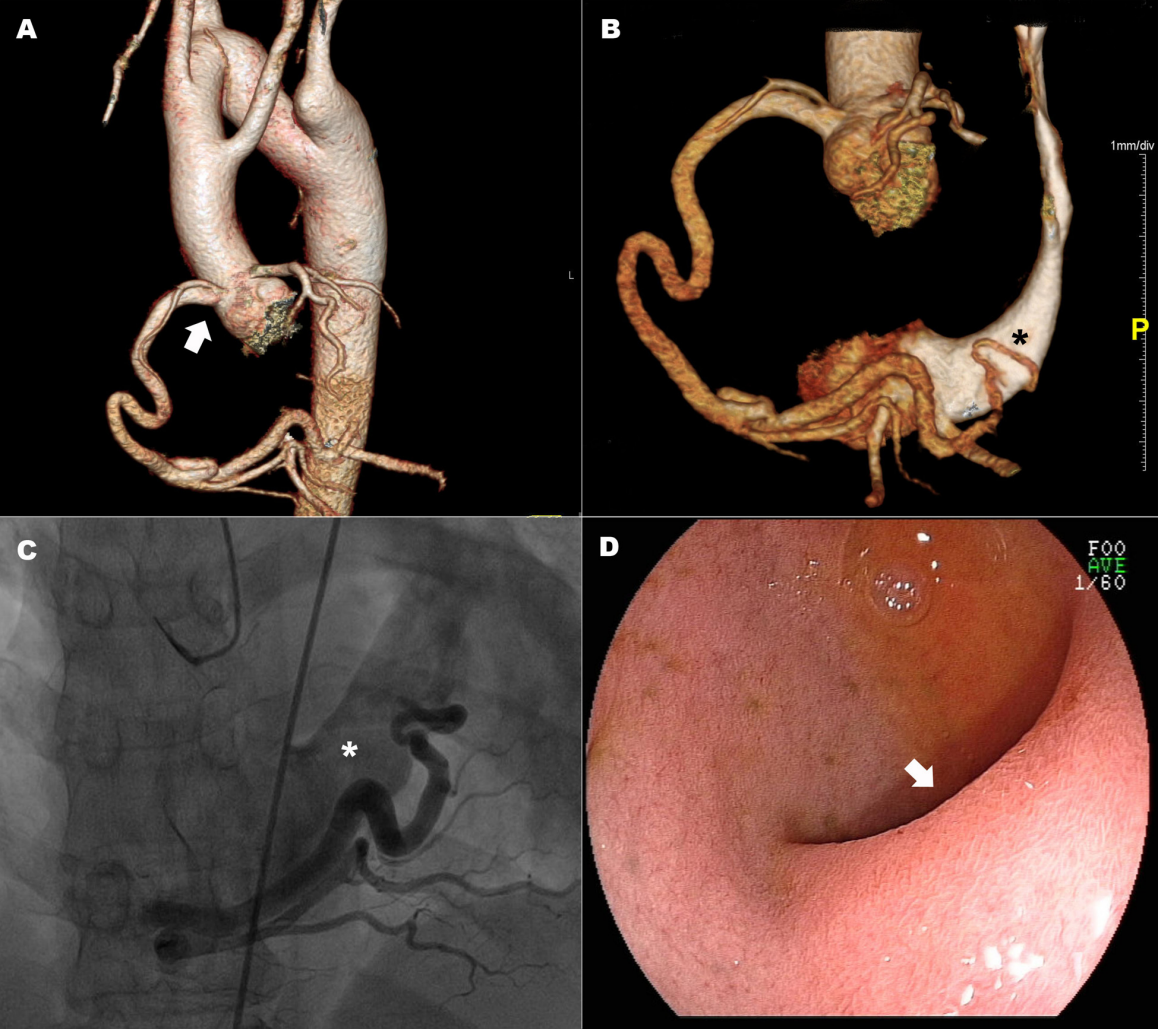
**Combined vascular anomalies of the right-sided aortic arch.** As evident in CT angiography, both coronary arteries arise above the sinus of Valsalva **(A, arrow)** and distal right coronary artery drains directly into the coronary sinus (B, *). The coronary artery-coronary sinus fistula was confirmed by angiography **(C)**. Esophagus was compressed by a Kommerell’s diverticulum as apparent in esophagogastroduodenoscopy **(D, arrow)**.

A 20-year old man was referred to the Department of Cardiology for assessment of a vascular abnormality incidentally detected in a CT scan obtained by the Otorhinolaryngology Department during pre-operative assessment for chronic otitis media. Other than otalgia, the patient reported no symptoms, including dysphagia or dyspnea. Physical examination revealed regular heart sounds without cardiac murmur. CT angiography with a 128-slice multi-detector scanner was performed for definitive diagnosis and comprehensive definition of thoracic anatomy. Multi-directional analysis of reconstructed 3D images of the thoracic aorta and its branches and of the trachea and esophagus (Figure 1) demonstrated that the trachea traveled inside the aortic ring and that the esophagus was between the distal aortic arch and a Kommerell’s diverticulum. Three large branches, left common carotid artery, right common carotid artery and right subclavian artery originated separately from the proximal aorta in said order. The left subclavian artery separately arose from a Kommerell’s diverticulum, a remnant of the left fourth aortic arch. In addition, the right and left coronary arteries aberrantly arose above the sinus of Valsalva (Figure 2A). The posterolateral branch of the right coronary artery drained directly into the coronary sinus (Figure 2B) as confirmed by coronary angiography (Figure 2C). Esophagogastroduodenoscopy showed that the esophagus was compressed by a pulsating external structure, namely the Kommerell’s diverticulum seen on CT angiography (Figure 2D). Aneurismal diameter was about 4.3 mm. Transthoracic echocardiography revealed an echo-free structure (1.38 × 1.55 cm sized) with abnormal continuous flow in the posterolateral side between left ventricle and atrium, that was suggested an enlarged coronary sinus with coronary fistula. But, another anomalies of heart were not detected. The patient had no symptoms related to the aforementioned vascular anomalies; careful follow-up for symptoms and aneurismal complications was recommended.

A right-sided aortic arch results from persistence of the right fourth aortic arch with suppression of the corresponding left vessels [4]. The classification of Felson and Palayew offers an anatomically sensible distinction between two variants clinically differing in presentation, associations, and prognosis [5]. Type 1, in which the left subclavian artery originates together with the left common carotid artery, or just proximal to it, is the mirror image of normal anatomy. In approximately 75% of these patients, cyanotic congenital anomalies (including tetralogy of Fallot, pulmonary stenosis with ventricular septal defects, tricuspid atresia, and truncus arteriosus) are present. Type 2 is associated with an aberrant left subclavian artery arising either as the last branch of the right-sided aortic arch or from an aortic diverticulum, known as a Kommerell’s diverticulum, which is a remnant of the left dorsal aortic arch. Type 2 is more common than type 1, and congenital heart anomalies are present in only 5% to 10%. The type 2 right-sided aortic arch is the variant form in the case presented here.

As previously mentioned, Kommerell’s diverticulum may cause symptoms of tracheal or esophageal compression, such as dysphagia, dyspnea, stridor, wheezing, cough, choking spells, or chest pain [3]. However, right-sided aortic arch may be asymptomatic [6]. Our patient did not experience symptoms related to compression even though the esophagus was compressed by the Kommerell’s diverticulum.

A right-sided aortic arch can present with a number of vascular anomalies of the aortic arch system [7]. In this patient, the right-sided aortic arch had multiple vascular variations including anomalous origin of left subclavian artery from a Kommerell’s diverticulum, supra-sinus origin of coronary arteries and coronary arteriovenous fistula. To the best of our knowledge, this is the first report of a patient with multiple vascular anomalies, such as coronary arteriovenous fistula in this setting.

Most symptoms requiring surgery are secondary to compression of the trachea or esophagus by the aberrant vascular structures [8]. Therefore, a meticulous analysis of vascular structure using 3D CT angiography is useful not only to define multiple combined vascular anomalies but also to demonstrate their relationships with the trachea and esophagus [9]. In addition, imaging with 3D reconstructions of the aorta can improve accuracy of diagnosis of multiple vascular anomalies and facilitate endovascular or surgical planning.

## Conclusions

Comprehensive imaging with 3D reconstructions could be the gold standard for detecting multiple vascular malformations and understanding relationships with adjacent structures in these patients.

## Consent

Written informed consent was obtained from the patient for publication of this case report and the accompanying images. A copy of the written consent form is available for review by the Editor of this journal.

## Abbreviations

CT: Computed tomography; 3D: Three-dimensional.

## Competing interests

The authors declare that they have no competing interests.

## Authors’ contributions

PJS conducted the clinical diagnosis, conceived the report and drafted the manuscript. SJW gathered patient data and helped to draft the manuscript. LCH wrote the case report and assisted in image acquisition and interpretation. Finally, PJS provided supervision and critically reviewed the manuscript. All authors gave their final acceptance to the submission of this report. All authors read and approved the final manuscript.

## Pre-publication history

The pre-publication history for this paper can be accessed here:

http://www.biomedcentral.com/1471-2261/14/104/prepub
